# A Phase 1b, Open-Label Study to Evaluate the Safety and Tolerability of the Putative Remyelinating Agent, Liothyronine, in Individuals with MS

**DOI:** 10.1007/s13311-023-01402-3

**Published:** 2023-07-17

**Authors:** Scott D. Newsome, Fan Tian, Thomas Shoemaker, Kathryn C. Fitzgerald, Sandra D. Cassard, Julie Fiol, Sarah Snoops, David S. Cooper, Jennifer S. R. Mammen, Pavan Bhargava, Ellen M. Mowry, Peter A. Calabresi

**Affiliations:** 1 Department of Neurology, Johns Hopkins University School of Medicine, Baltimore, MD USA; 2 Division of Neuroimmunology and Neurological Infections, Department of Neurology, Baltimore, MD USA; 3 Department of Mathematics, Tufts University, Medford, USA; 4 Department of Neurology, Rush University, Chicago, IL USA; 5 National Multiple Sclerosis Society, New York, NY USA; 6 Department of Medicine, Division of Endocrinology, Johns Hopkins University School of Medicine, Baltimore, MD USA

**Keywords:** Multiple sclerosis, Remyelination, Thyroid hormone, Clinical trial

## Abstract

Thyroid hormones are essential during developmental myelination and may play a direct role in remyelination and repair in the adult central nervous system by promoting the differentiation of oligodendrocyte precursor cells into mature oligodendrocytes. Since tri-iodothyronine (T3) is believed to mediate the majority of important thyroid hormone actions, liothyronine (synthetic T3) has the potential to induce reparative mechanisms and limit neurodegeneration in multiple sclerosis (MS). We completed a phase 1b clinical trial to determine the safety and tolerability of ascending doses of liothyronine in individuals with relapsing and progressive MS. A total of 20 people with MS were enrolled in this single-center trial of oral liothyronine. Eighteen participants completed the 24-week study. Our study cohort included mostly women (11/20), majority relapsing MS (12/20), mean age of 46, and baseline median EDSS of 3.5. Liothyronine was tolerated well without treatment-related severe/serious adverse events or evidence of disease activation/clinical deterioration. The most common adverse events included gastrointestinal distress and abnormal thyroid function tests. No clinical thyrotoxicosis occurred. Importantly, we did not observe a negative impact on secondary clinical outcome measures. The CSF proteomic changes suggest a biological effect of T3 treatment within the CNS. We noted changes primarily in proteins associated with immune cell function and angiogenesis. Liothyronine appeared safe and was well tolerated in people with MS. A larger clinical trial will help assess whether liothyronine can promote oligodendrogenesis and enhance remyelination in vivo, limit axonal degeneration, or improve function.

## Introduction

Multiple sclerosis (MS) is a chronic, immune-mediated disease of the central nervous system (CNS) that is characterized by inflammation, demyelination, and neurodegeneration [1]. It remains the most common non-traumatic cause of neurologic disability in young adults and presents in most patients as relapsing–remitting disease [2]. Relapses, caused by inflammatory demyelination, can result in a significant amount of neurological disability and reduced health-related quality of life, and having frequent early relapses is associated with increased risk of longer-term disability [3–6]. Clinical recovery from early relapses is incomplete in approximately half of patients with MS [7]. The mechanisms underlying relapse recovery are not completely understood.

Remyelination of acutely denuded axons is one mechanism by which relapse recovery may occur. Remyelination may occur via newly differentiated oligodendrocytes, which are derived from oligodendrocyte precursor cells (OPCs) in the CNS. However, despite the presence of this innate repair mechanism, many patients later develop progressive functional disability. This may be due to a failure of remyelination or because of progressive axonal injury. Chronic demyelinating lesions are surrounded by OPCs and premyelinating oligodendrocytes, which suggest that failed remyelination does occur and could be partially due to incomplete oligodendrocyte differentiation [8]. Additionally, studies have highlighted the importance of mitochondrial dysfunction, perhaps related to oxidative stress or increased energy demands, in mediating MS disease progression [9, 10]. Mitochondrial dysfunction may drive axonal degeneration with resultant neurodegeneration and progressive neurological decline (progressive MS) [10]. While numerous immune modulating therapies exist, currently, there is an urgent need for novel therapies that have neuroreparative and neuroprotective properties.

During development, thyroid hormones are essential for the development of the nervous system through enhancing neurogenesis, synaptogenesis, and glial cell differentiation promoting myelination. Hence, thyroid hormones may play a direct role in remyelination and repair in the adult CNS by promoting maturation of oligodendrocytes. Furthermore, thyroid hormones have been shown to reduce oxidative stress and thus may have the capacity to prevent mitochondrial dysfunction as well. In mice, tri-iodothyronine (T3) administration has been shown to help facilitate recovery from cuprizone-induced demyelination [11]. Since T3 is believed to mediate the majority of important thyroid hormone actions, liothyronine (synthetic form of T3) has the potential to induce reparative mechanisms and limit secondary neurodegeneration in MS. The main goal of this phase 1b study was to assess the safety and tolerability of ascending doses of liothyronine in individuals with MS. Symptoms and signs of hyperthyroidism could occur in people taking T3, so specific pre-planned assessments with interventions have been incorporated into our study design. Secondary goals were to evaluate if liothyronine administration has effects on clinical disability measures and/or health-reality quality of life measures.

## Methods

### Overall Study Design and Participation

This was a phase 1b, single center open-label dose escalation study that was approved by the Johns Hopkins Medicine Institutional Review Board. All participants provided informed consent before enrolling. The clinical trial was registered on clinicaltrials.gov (NCT02506751) and the Food and Drug Administration (FDA) determined that this study met all requirements for exemption from investigational new drug (IND) regulations.

**Fig. 1:**
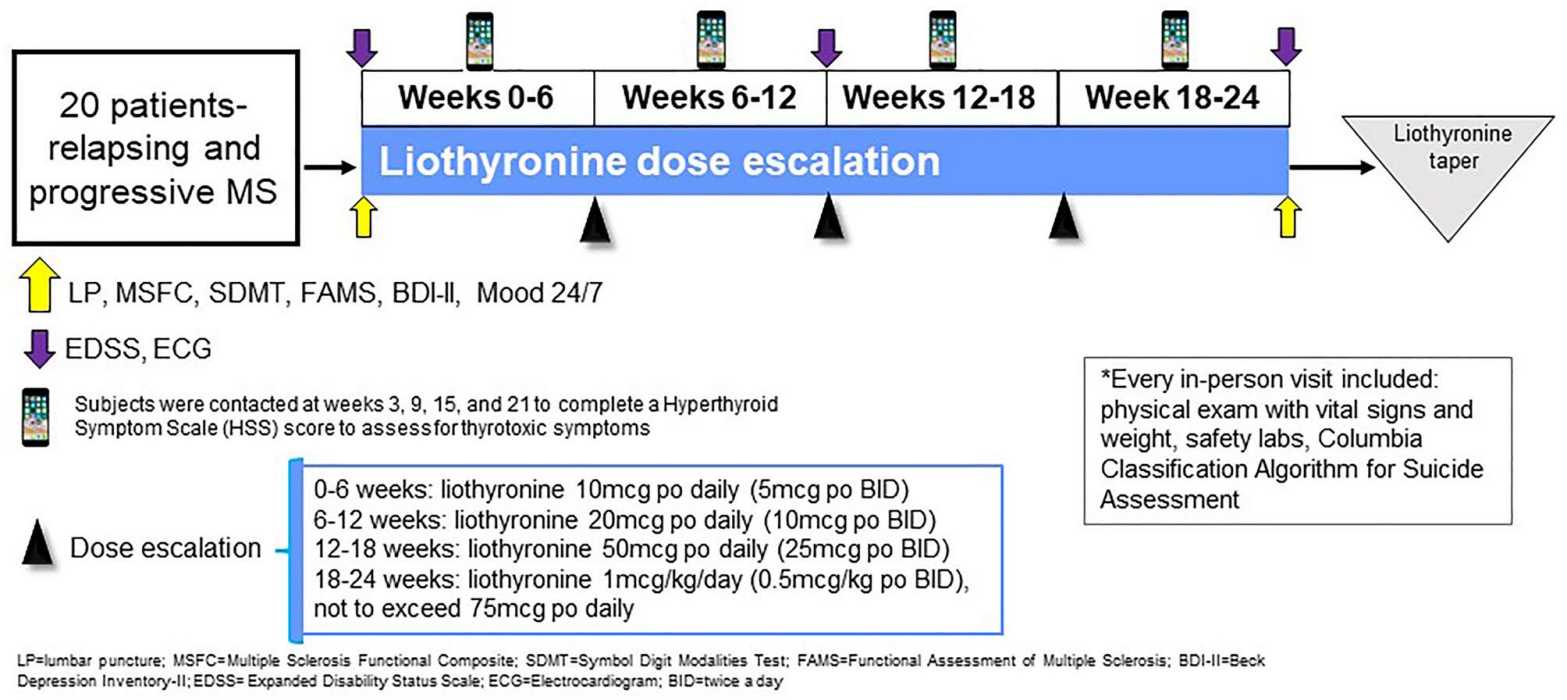
Study design

**Fig. 2:**
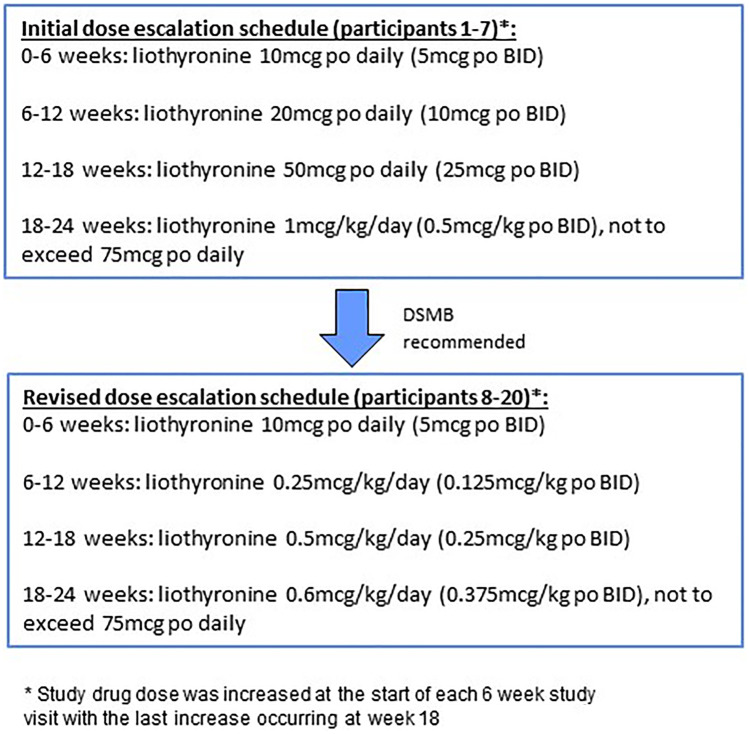
Change in liothyronine dose escalation schedule

A convenience sample of 20 patients with MS was enrolled in the study after meeting the inclusion/exclusion criteria (see Table 1). The study consisted of a screening and baseline visit, followed by visits every 6 weeks for the 24-week duration of the study in order for participants to receive their study drug and to monitor drug safety and tolerability (see Fig. 1). Safety and clinical assessments were obtained throughout the study as outlined below. All eligible participants were treated with the study drug, liothyronine, as per the standardized dose-escalation protocol (not to exceed 75mcg daily, see Fig. 1). The study drug dose was increased at the start of each 6-week study visit with the last increase occurring at week 18 (Fig. 2). The mean and maximum total daily liothyronine doses reached were 48mcg and 75mcg, respectively. The study drug was procured, handled, and dispensed by our internal Investigational Drug Services pharmacy.

**Table 1:** Study inclusion and exclusion criteria

**Inclusion criteria**
1. Must meet 2010 McDonald criteria for clinically definite MS
2. Age 18 to 58 years
3. Must be euthyroid
4. EDSS 3.0–7.5
5. Patients may be on MS immunomodulating therapies or immunosuppressant therapies during the study
**Exclusion criteria**
1. Known thyroid disease (past or current)
2. Currently on thyroid replacement therapy
3. Steroid use within a month of screening
4. History of coronary artery disease, atrial fibrillation, or other clinically significant cardiac disease
5. History of adrenal insufficiency
6. Ongoing renal and/or liver disease
7. Ongoing severe depression and/or anxiety
8. Use of carbamazepine, phenytoin, phenobarbital, warfarin, antacids, cholestyramine, colestipol, sucralfate, and rifampin^a^
9. Known contraindication to using beta-blocker medications
10. History of alcohol or substance abuse in the past 6 months
11. Pregnant or nursing
12. Investigator feels that participation in this study is not in the best interest of the subject

^a^These are common Cytochrome P450 (CYP450) Inducers which could reduce the concentration of liothyronine as this medication is metabolized via the CYP450 system

Medication adverse event visits, premature withdrawal visits, and unscheduled visits for relapses were conducted as needed.

### Safety Assessments

During the screening visit, participants had lab work done (thyroid stimulating hormone [TSH], comprehensive metabolic panel, urine pregnancy test) to assess for study eligibility criteria. Serum thyroid function tests (TSH, free thyroxine [T4], and T3) were repeated at every study visit moving forward. Participants also underwent blood chemistry testing at weeks 6, 12, 18, and 24.

The following pre-defined study drug dosing algorithm was used if thyroid dysfunction (clinical or bio-chemical) was detected during the study: (A) symptoms of anxiety, tremor and insomnia led to a dose reduction to former dose irrespective of the serum T3 level. If symptoms persisted 1 week after dose reduction, another dose reduction was implemented; (B) palpitations were evaluated with an electrocardiogram (ECG) to assess for rhythm abnormalities and then managed with a dose reduction regardless of ECG findings. If symptoms persisted after 1 week, another dose reduction was done; and (C) if a subject’s serum TSH dropped to < 0.5 mU/L and/or serum T3 was greater than 10% above the upper limit of normal, the study drug dose was reduced to the subject’s previously-administered dose. Additional pre-defined monitoring for adverse effects (AEs) was performed throughout the study including assessing vital signs (pulse rate, blood pressure, and temperature), weight, and urine pregnancy test every 6 weeks, and subjects completed a hyperthyroid symptom scale (HSS) score to assess for thyrotoxic symptoms at weeks 3, 9, 15, and 21. The HSS score was collected between the in-person study visits for additional safety monitoring [12]. Subjects were also contacted after they tapered off liothyronine in order to assess difficulties with the wean, and an unscheduled study visit was performed if withdrawal issues occurred.

In compliance with the FDA regulations on assessing for suicidality in prospective trials, the Columbia Classification Algorithm for Suicide Assessment was administered at all in-person study visits.

All safety data were recorded per International Conference on Harmonization (ICH) Guideline for Good Clinical Practice E6(R1) and all AEs were graded based on the National Cancer Institute’s Common Terminology Criteria for AEs (version 4.0). In order to adhere to the ICH guidelines for reporting serious adverse events (SAE), the IRB was notified within 24 hours of the study staff becoming aware of a SAE.

The study drug was to be permanently discontinued for one or more of the following pre-defined reasons: (A) if a participant developed intolerable thyrotoxic symptoms, (B) if a participant’s thyroid function tests remain abnormal despite dose reduction of study drug, (C) if the participant became pregnant or suicidal, (D) if the participant experienced a grade 3 or higher AE that was reported as possibly related to the study drug, and (E) if the participant or treating physician wanted to prematurely withdraw them from the study.

The data safety monitoring board consisted of two endocrinologists (JSRM and DSC) and one neurologist (EMM). The DSMB met on a predetermined schedule (every 6 months until completion of the trial) and any time an SAE occurred.

### Clinical Monitoring Measures

Clinical measures were obtained to assess for changes in clinical and patient-reported outcome measures during the course of the study (see Fig. 1). Specifically, these measures were being done to monitor for signs and/or symptoms associated with a negative impact of liothyronine on MS. Overall disability was measured by the Expanded Disability Status Scale (EDSS) and multiple sclerosis functional composite (MSFC) scores: EDSS was administered at the screening, week 12, and end of study visits; MSFC was administered at the screening, baseline, and end of study visits. A participant was considered to have progression of disability if there was an increase in their EDSS score by at least 1.0 point at the end of the study or worsening of the score of at least one MSFC component by 20% or more at the end of the study [13]. The Symbol Digit Modalities Test (SDMT) and Functional Assessment of Multiple Sclerosis (FAMS) were administered at the baseline and end of study visits [14]. Mood was assessed with the Beck Depression Inventory II (BDI-II), a 21-item self-report instrument for measuring the severity of depression. The BDI-II was administered at the screening and end of study visits. Mood 24/7 was used to record participants’ daily mood (via “Mood24/7”; http://www.mood247.com); this is a free service that texts mobile phones daily asking to rate participant’s moods on a scale of 1 (low) through 10 (high). Subject’s Mood 24/7 entries were assessed at the final visit, when we received a printout of the course of participant’s mood during the study. Participants started recording their daily mood at the screening visit.

### Exploratory Biomarkers

Serum and cerebrospinal fluid (CSF) were collected at baseline and end of study in order to assess liothyronine’s impact on neurotrophic/neuroprotective markers. SOMAscan platform (DNA aptamer based detection of proteins) was used to detect and quantify a panel of 1314 proteins in the CSF. 

### Statistical Analysis

Descriptive and summary statistics for actual values and change from baseline for the primary safety outcome are shown where appropriate. Safety data was captured in real time, and assessments were focused on AEs (including study treatment tolerability assessments, laboratory evaluations, vital signs, and physical examination) and reported as such. The incidence rate of AEs was recorded by system organ class, severity, and by relationship to the study treatment. Overall summary of relevant AEs was reported if occurrence was greater than 10% of participants. Tolerability analysis was based on the number (%) of subjects who failed to complete the study due to adverse events. SAEs are reported using descriptive statistics.

We assessed safety and tolerability of liothyronine in longitudinal analyses using mixed-effect regression models. Our initial analysis examined changes in serum thyroid function test scores and in measurements of EDSS, timed walking tests and health-related quality of life. Parameters of serum test scores, timed 25-foot walk test (T25FW) and nine-hole peg test (9HPT) were log-transformed to meet the normality assumption of the model. We further analyzed the changes in the main outcomes after adjusting for relevant characteristics (age, gender, BMI) as well as MS disease duration. We also considered the effect on the outcomes between the two different disease subtypes (relapsing and progressive MS), and we additionally fitted models after adjusting for the interaction of disease subtype with time. The correlations were examined by using analysis of variance (ANOVA) method. We performed subgroup analyses using similar models to describe the changes for each of the disease subtypes.

We used a Wilcoxon Rank Sum test to determine if CSF proteins changed significantly over the course of the study. We used PANTHER and STRING databases to perform pathway enrichment analyses to determine the functional relevance of the changes in the proteome. No specific adjustments were made prior to the CSF analysis since the data analyzed was assessing protein changes at the individual level (within participant).

## Results

### Baseline Demographics and Clinical Characteristics

Study participants were recruited from the Johns Hopkins MS Center between 8/28/2015 and 3/22/2017. A total of 22 patients with MS were screened with one screen failure noted due to an abnormal screening TSH and another screen failure due to ongoing severe depression. Twenty eligible patients with MS enrolled in the study, 18 of whom completed the study. Enrolled participants included; mostly woman (11/20), majority relapsing MS (12/20), mean age 46, and screening median (interquartile range) for EDSS was 3.5 (3.5–6.0). See Table 2 for the full details of our study cohorts’ baseline characteristics. Relevant baseline safety and clinical assessments are in Table 3.

**Table 2:** Participants baseline demographics and clinical characteristics

	**Baseline** **(Week 0)**
***N***^**a**^	20
**Age, years, mean (SD)**	46.4 (8.5)
**Female sex, *n* (%)**	11 (55%)
**Race, *n* (%)**	
**Black**	4 (20%)
**White**	15 (75%)
**Other**	1 (5%)
**Body mass index, kg/m**^**2**^**, mean (SD)**	26.7 (5.0)
**Age at first symptom, years, mean (SD)**	34.5 (8.1)
**Age at diagnosis, years, mean (SD)**	36.6 (8.5)
**Disease duration, years, mean (SD)**	9.8 (5.6)
**Disease subtype**	
Relapsing–remitting, *n* (%)	12 (60%)
Progressive, *n* (%)	8 (40%)
**Multiple sclerosis disease modifying therapy, *n* (%)**	17 (85%)
Glatiramer acetate, 1	
Dimethyl Fumarate, 4	
Fingolimod, 2	
Natalizumab, 7	
Rituximab, 3	
**EDSS, median, (IQR)**	3.5 (3.5–6.0)

*N* sample size, *SD* standard deviation, *kg/m*^*2*^ kilograms per meter squared, *EDSS* Expanded Disability Status Scale, *IQR* Interquartile range

^a^Screened 22 with 2 screen failures: 1 abnormal TSH and 1 severe depression

**Table 3:** Relevant baseline safety and clinical assessments

**Serum thyroid function tests**	
Thyroid stimulating hormone, median (Q1-Q3)	1.7 (1.3–2.4)
tri-iodothyronine [T3], median (Q1-Q3)	1.1 (1.0–1.2)
free thyroxine [T4], median (Q1-Q3)	1.2 (1.2–1.3)
**Hyperthyroid symptom scale [HSS] score, median (Q1-Q3)**	8.0 (3.50–8.0)
**ECG, abnormal-not clinically significant**^**a**^**, *n* (%)**	5 (0.25)
**MSFC components**	
25-foot walking speed, median (Q1-Q3)	7.1 (5.5–17.5)
PASAT, median (Q1-Q3)	52.5 (46.5–58.0)
9-hole peg test, median (Q1-Q3)	24.6 (22.1–32.0)
**SDMT, median (Q1-Q3)**	53.0 (44.0–57.0)
**Overall FAMS, median (Q1-Q3)**	121.5 (104.8–142.0)

*MSFC* multiple sclerosis functional composite, *ECG* electrocardiogram, *PASAT* paced auditory serial

Addition test: *SDMT* Symbol Digit Modalities Test, *FAMS* Functional Assessment of Multiple Sclerosis

^a^Incomplete bundle branch block, sinus tachycardia, non-specific T-wave changes, possible left atrial abnormalities, sinus bradycardia

### Overall Safety and Tolerability

The majority of study participants completed the trial (18/20, 90%), and there were no deaths. Only one participant self-discontinued the study drug after their week 18 visit due to treatment-emergent AE that was deemed possibly related to study drug. The participant developed bowel incontinence and abdominal pain which resolved after stopping the study drug. Notably, this participant had a prior history of diverticulitis and a long-standing history of irritable bowel syndrome.

**Fig. 3:**
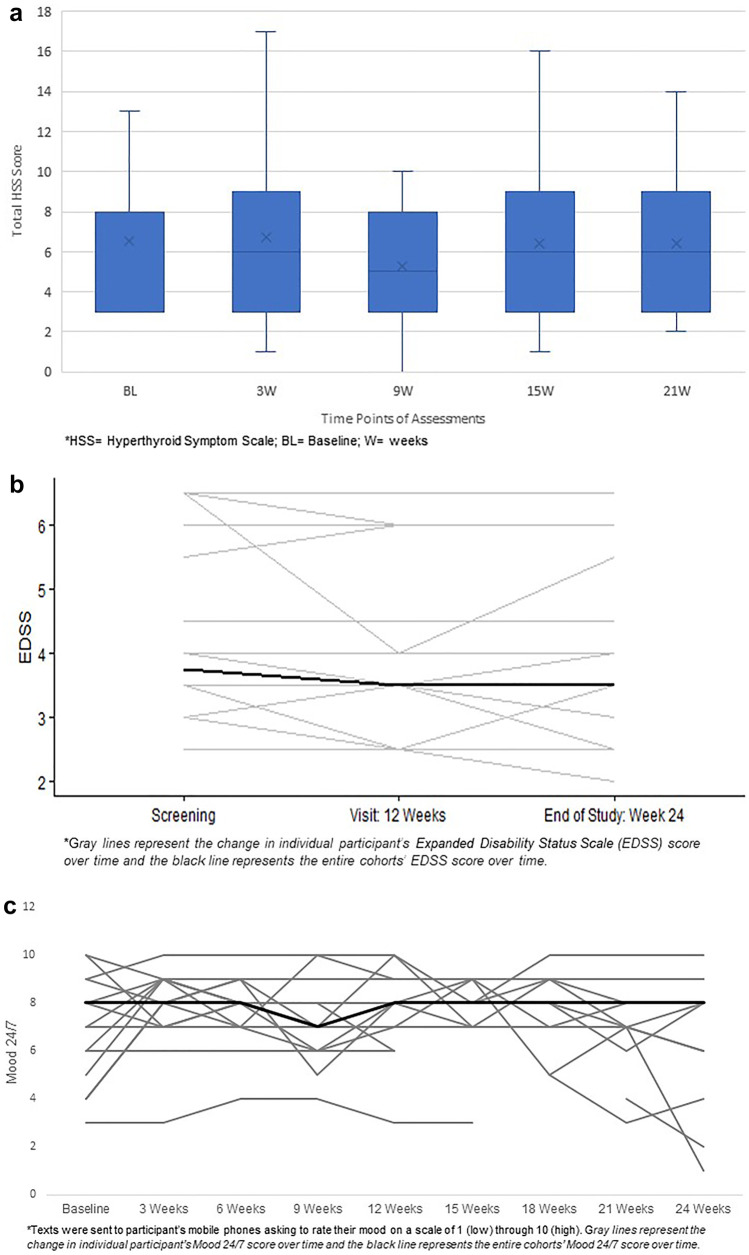
**a** Impact of liothyronine on Hyperthyroid Symptom Scale; **b** Impact of Liothyronine on Expanded Disability Status Scale; **c** Impact of Liothyronine on Mood 24/7

The most common relevant AEs reported during the study (in order of prevalence) included gastrointestinal distress, fatigue, headaches (HAs), insomnia, and palpitations. The majority of these AEs were grade 1 and self-limiting without intervention even in the setting of continuing the study drug and none occurred during the wean off time period. Importantly, the ECGs of participants were stable throughout the study and required no dose adjustment of the study drug. Additionally, the aforementioned participant with bowel incontinence took anti-diarrheal medications and was reported as a grade 2 AE. The full details of the overall summary of relevant AEs reported based on number of events and unique patients associated with these events are in Table 4. There were only two SAEs reported throughout the duration of the study, neither of which were thought to be study drug related (Table 5). In addition, the HSS score did not change throughout the study for participants (Fig. 3a), and no unscheduled visits were required for clinical thyrotoxicosis.

**Table 4:** Overall summary of relevant adverse events (occurring > 10%)

**Adverse Event**	**Number of events**^**a**^	**Number of unique patients**
**Any adverse event**	255	
**Most common adverse events associated with treatment**		
Gastrointestinal^b^	39	9
Endocrine/Metabolic		
TSH	8	8
T3	5	4
Headache	19	7
Fatigue	26	7
Insomnia	12	5
Tachycardia/palpitations	3	3

^a^Individual symptoms counted each time reported (most Grade 1 and self-limiting; one participant had Grade 2 for bowel incontinence)

^b^loose stool/diarrhea, flatulence, appetite changes, bowel urgency/incontinence, abdominal pain, constipation, nausea, vomiting, heartburn, GI upset

**Table 5:** Serious adverse events reported

**Participant**	**Weeks into study**	**Related to Intervention**	**Outcome**	**Description of serious adverse events**
9	13	No	Resolved	Hospitalization for urinary tract infection
15	6	No	Surgery, chemotherapy, and radiation	Hospitalization for severe back pain; found to have lumbar stenosis due to plasmacytoma

No relapses or disability progression occurred during the study. One participant changed their disease modifying therapy at week 19 due to preference in route of administration (fingolimod to ocrelizumab).

### Impact on Safety Lab Monitoring and Study Drug Regimen

Serum thyroid function tests did change over time but did not result in clinical thyrotoxicosis. Four out of the initial 7 study participants had to reduce T3 dose over the course of the study because the initial study protocol mandated that this would be needed in the context of an evolving bio-chemical thyrotoxicosis (asymptomatic low TSH [< 0.5 mU/L]). In response to this common occurrence, the DSMB recommended changing the dose escalation schedule (see dosing schedule chart, Fig. 2) and to only decrease the study drug dose if the participant’s TSH was < 0.5 mU/L and was associated with hyperthyroid symptoms (clinical thyrotoxicosis). No other changes were made to the study drug dosing algorithm for emerging thyroid dysfunction (bio-chemical or clinical), since other pre-defined safety measures were not impacted as defined by the study protocol.

Participants 8 through 20 did not require a dose reduction after the revised dose escalation schedule was implemented. Moreover, fewer systemic side effects were observed with the new dose escalation regimen. Specifically, the gastrointestinal AEs decreased to 33% from 70% and HAs decreased to 8% compared to 86%.

### Impact on Secondary Clinical Outcome Measures

The EDSS remained stable throughout the study (Fig. 3b) for the entire cohort as did the SDMT, MSFC, FAMS, and Mood 24/7 (Fig. 3c).

In the pre-planned subgroup analyses, the majority of clinical measures were unchanged from baseline to end of study for both relapsing (*n* = 12) and progressive MS (*n* = 8) patients. The T25FW slightly improved in progressive patients and there was a trend for improved thinking/fatigue on FAMS in relapsing patients (Tables 6 [relapsing MS] and 7 [progressive MS]).

**Table 6:** Change in clinical measures from baseline to end of study in relapsing–remitting MS

**Outcomes**		**Results of multivariate regression model**^a^	
***n* = 12**		Estimate (± s.d.)	*p*-value
**EDSS**		− 0.29 (0.19)	0.17
**(log) T25fw**		0.021 (0.044)	0.65
**(log) 9HPT**		0.015 (0.022)	0.51
**SDMT**		− 1.42 (2.10)	0.52
**PASAT**		0.79 (1.20)	0.53
**FAMS**	Total	3.76 (5.12)	0.48
	Mobility	0.98 (1.12)	0.41
	Symptoms	0.20 (0.94)	0.84
	Thinking and fatigue	3.64 (1.66)	0.06
	General contentment	− 0.22 (1.33)	0.87
	Emotional well-being	0.15 (1.11)	0.89
	Social well-being	− 0.53 (0.64)	0.43

*EDSS* Expanded Disability Status Scale, *MSFC* multiple sclerosis functional composite, *T25fw* timed 25 foot walk test, *9HPT* nine-hole peg test, *SDMT* Symbol Digit Modalities Test, *PASAT* paced auditory serial addition test, *FAMS* Functional Assessment of Multiple Sclerosis

^a^Each outcome were adjusted for age, sex, body mass index, and MS duration for analysis. Higher scores on the FAMS indicate a better quality of life and numbers shown in table are the adjusted numbers from the multivariate analysis

**Table 7:** Change in clinical measures from baseline to end of study in progressive MS

**Outcomes**		**Results of multivariate regression model**^**a**^	
***n* = 8**		Estimate (± s.d.)	*p*-value
**EDSS**		− 0.024 (0.12)	0.85
**(log) T25fw**		− 0.33 (0.063)	0.01
**(log) 9HPT**		− 0.011 (0.046)	0.82
**SDMT**		2.40 (1.26)	0.12
**PASAT**		− 1.53 (1.64)	0.39
**FAMS**	Total	1.40 (3.37)	0.69
	Mobility	0.78 (0.97)	0.46
	Symptoms	0.83 (1.74)	0.65
	Thinking and fatigue	− 1.21 (1.88)	0.55
	General contentment	− 0.59 (0.96)	0.57
	Emotional well-being	1.16 (1.37)	0.43
	Social well-being	0.18 (1.01)	0.87

*EDSS* Expanded Disability Status Scale, *MSFC* multiple sclerosis functional composite, *T25fw* timed 25 foot walk test, *9HPT* nine-hole peg test, *SDMT* Symbol Digit Modalities Test, *PASAT* paced auditory serial addition test, *FAMS* Functional Assessment of Multiple Sclerosis

^a^Each outcome were adjusted for age, sex, body mass index, and MS duration for analysis. Higher scores on the FAMS indicate a better quality of life and numbers shown in table are the adjusted numbers from the multivariate analysis

### Impact on Exploratory CSF Biomarkers

CSF was collected at baseline and end of study (24 weeks) as an exploratory outcome for treatment response in 16 patients. A proteomics platform was used to assess the effect of liothyronine treatment on the CSF proteome in MS.

**Fig. 4:**
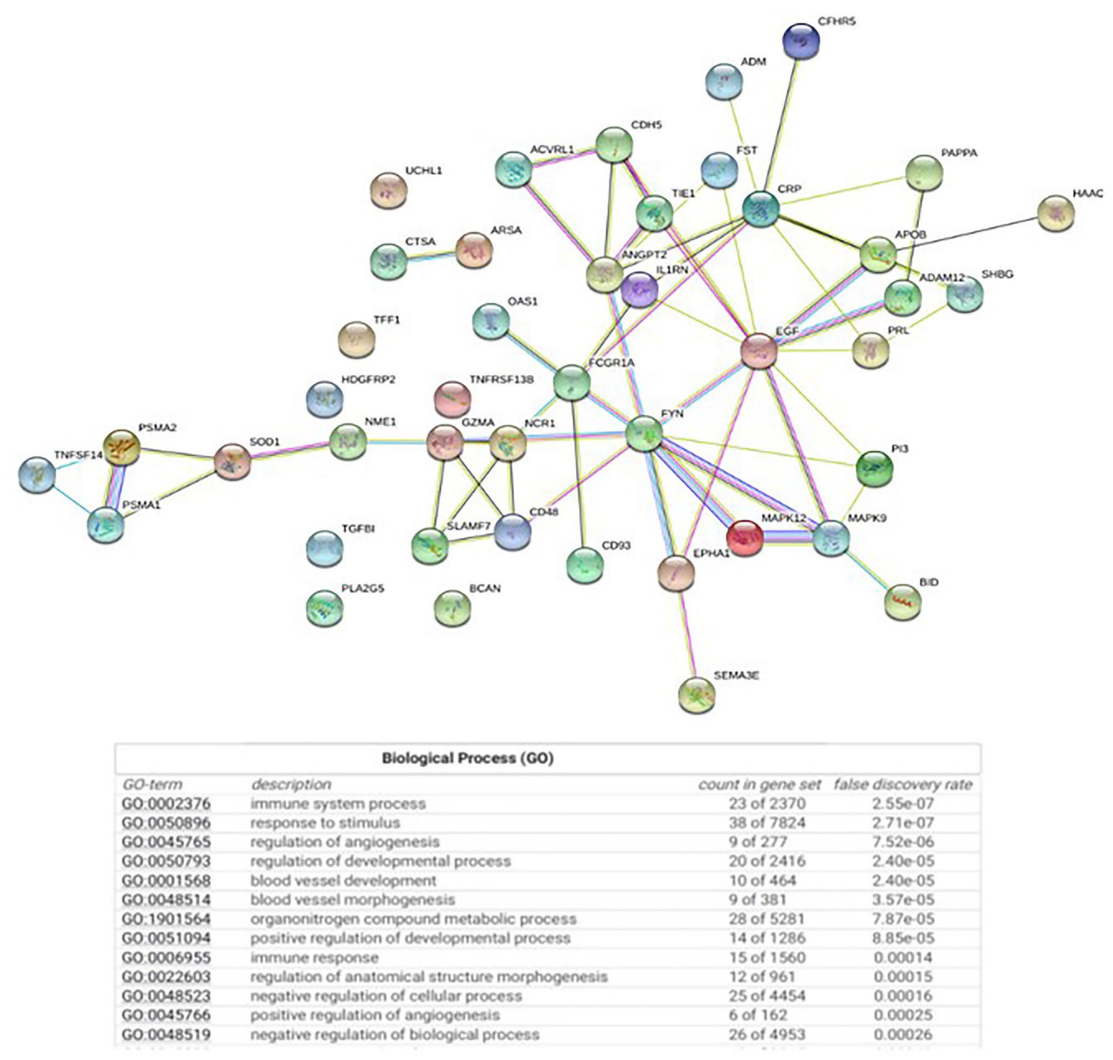
Enrichment analyses revealed over-represented angiogenesis and innate and adaptive immune function

Of the measured proteins, 46 changed (19 increased and 27 decreased) over the course of the study (*p* < 0.05). These included proteins related to immune function such as TACI, NKp46, IgA, and IgD and angiogenesis such as Cadherin-5, sTIE-1, and ANGPT2. Enrichment analyses using PANTHER and STRING databases revealed that the biological processes that were over-represented included angiogenesis and innate and adaptive immune function (Fig. 4). Angiogenesis-related proteins predominantly demonstrated an increase with liothyronine treatment and the majority of immune-related proteins decreased with treatment (Table 8).

**Table 8:** Top proteins altered by liothyronine treatment

**Protein symbol**	**Name**	**Log2 fold change**	**Direction of change**	***p*-value**
Cadherin-5	Vascular endothelial cadherin	0.13	Increased	4.20E-04
SHBG	Sex hormone binding globulin	0.35	Increased	0.002
TACI	Transmembrane activator and CAML interactor	− 0.12	Decreased	0.002
Elafin	Peptidase inhibitor 3	− 0.21	Decreased	0.002
NKp46	Natural cytotoxicity triggering receptor 1	− 0.10	Decreased	0.004
sTie-1	Tyrosine kinase with Ig and EGF homology domains 1	0.15	Increased	0.004
IgA	Immunoglobulin A	− 0.27	Decreased	0.005
IgD	Immunoglobulin D	− 0.50	Decreased	0.005
NME1	Nucleoside diphosphate kinase A	− 0.20	Decreased	0.005
PLA2G5	Phospholipase A2 group V	0.33	Increased	0.006
PAPP-A	Pregnancy-associated plasma protein A	0.11	Increased	0.009
MAPK9	Mitogen-activated protein kinase 9	0.08	Increased	0.010
EphA1	Ephrin A1	0.06	Increased	0.012
TFF1	Trefoil factor 1	0.06	Increased	0.013
CFHR5	Complement factor H related protein 5	0.15	Increased	0.014
SOD	Superoxide dismutase	− 0.10	Decreased	0.016
SLAF7	SLAM family member 7	− 0.35	Decreased	0.016
IL-1Ra	Interleukin-1 receptor a	− 0.07	Decreased	0.018
OAS1	2′-5′-oligoadenylate synthase 1	0.06	Increased	0.023
BGH3	Transforming growth factor-beta-induced protein ig-h3	0.11	Increased	0.040

## Discussion

This phase 1b open-label, dose-escalation clinical trial in people with MS demonstrated that liothyronine was well-tolerated without treatment-related severe and/or serious adverse events. In fact, the majority of participants completed the trial (90%). Moreover, there was no evidence of MS disease activation or clinical deterioration for the duration of the study. Similar tolerability and trial adherence were seen in another phase 1 trial with liothyronine, which reinforces its possible use as an add-on therapy in MS with the treatment goals of remyelination and neuroprotection in people with MS [15].

It is well-established that thyroid hormones are critical for brain development, including myelination. Every cell and organ system, including the CNS, is influenced by thyroid hormones (T4 and T3) [16]. During development, thyroid hormones bind to thyroid hormone receptors alpha and beta, allowing nuclear translocation and altered gene transcription, which facilitates maturation of oligodendrocytes and enhances myelination [17]. In addition, other processes associated with terminal brain differentiation are regulated by thyroid hormones, including dendritic and axonal growth, synaptogenesis, and neuronal migration [17]. In adults, thyroid hormone is important for neuronal health and remyelination. While these developmental roles of thyroid hormones are well known, thyroid hormones may also play a critical role in the adult CNS not only for maintenance of tissues, but also during remyelination and repair [18]. In addition, thyroid hormones may help prevent oxidative stress in astrocytes via maintaining glutathione homeostasis, which may be critical for protecting neurons [19]. Moreover, thyroid hormone plays an essential role in decreasing mitochondrial stress, which may limit apoptosis and facilitate cellular reparative processes [20–22].

Since thyroid hormones appear to play a role in remyelination and axon preservation within the CNS, processes that are affected by MS, thyroid hormones represent worthwhile candidates for putative neuroreparative and neuroprotective trials. Specifically, T3 analogs appear to be the most promising agents because T3 is believed to mediate the most important thyroid hormone actions. Hence, liothyronine was chosen as the candidate agent for our study. Of course, it is critical to establish that a T3 analog is safe and tolerable to use in MS especially in euthyroid patients who are on MS DMTs.

In our study, the most common adverse events included gastrointestinal distress and abnormal thyroid function tests, although no clinical thyrotoxicosis occurred or was unmasked over time. The gastrointestinal symptoms were self-resolving except for one participant who decided to stop the study medication. As previously mentioned, this participant had a prior history of irritable bowel syndrome and diverticulitis, highlighting that thyromimetics might not be appropriate for patients with pre-existing bowel disorders/diseases. On the other hand, weight-based dosing earlier in the course of treatment may also help minimize side effects, as was observed after the dosing regimen was revised in our study (Fig. 2).

The use of T3 in euthyroid subjects has been studied outside of MS and was found to be safe, including at higher doses than our study. The randomized clinical trials using T3 supplementation in euthyroid patients with medication-resistant depression have generally been short term and have used a wide range of doses. These studies have shown preliminary efficacy in ameliorating refractory depression, suggesting that T3 may impact on CNS processes. While there is limited information on the effects on bio-chemical markers of thyroid function or the rates of thyrotoxic symptoms in patients, high rates of significant adverse effects have not been reported [23]. A recent case review study of 159 patients with treatment-resistant bipolar disease who used T3 augmentation therapy at an average dose of 90.4 mcg (range; 13–188mcg) also reported low rates of adverse effects. The most common adverse effect was tremor, which responded to T3 dose reduction. Only one patient with a prior history of atrial fibrillation experienced recurrence of the arrhythmia while on 125mcg of T3 supplementation. The patient was treated successfully with a change in her medication regimen, including T3 dose reduction [23]. Overall, the experience from these studies suggests that T3 supplementation in appropriately chosen euthyroid patients is well tolerated.

Even though multiple studies have now shown T3 administration to be generally tolerable and safe, a selective thyroid receptor-beta thyromimetic may be preferred in patients with certain co-morbidities. A selective thyromimetic could prove to be more efficacious and better tolerated since there should be fewer systemic side effects when thyroid receptor-alpha is not targeted [24]. However, there is a lack of comparative data showing a clinically meaningful difference between non-selective and selective thyromimetics.

While our study was a phase 1b safety and tolerability study, we were interested in assessing whether there were trends for improvement in clinical measures, since T3 administration has shown the potential to induce reparative mechanisms and possibly limit secondary neurodegeneration in MS [11, 25–27]. We noted a change in the T25FW in progressive patients and trend for improved thinking/fatigue in relapsing patients on the SF36. Although these changes may be of interest, they need to be interpreted with caution due to the small sample size, short duration of trial, lack of comparator group, and open-label nature of the study. Moreover, type 1 errors can be inflated in certain small group analyses and we do not want to underestimate the potential for an endocrine effect on the clinical measures versus a neuroprotective or remyelinating effect. Importantly, we did not observe a negative impact on other clinical measures (e.g., EDSS, MSFC, Mood 24/7) which could have occurred despite T3’s putative mechanism of action in MS as was seen in prior clinical trials using cytokine modulators, Lenercept and Infliximab, and a peptide analog of human myelin basic protein, Tiplimotide [28].

The CSF proteomic changes suggest a biological effect of T3 treatment within the CNS. We noted changes primarily in proteins associated with immune cell function and angiogenesis. Even though these findings are keeping with known functions of thyroid hormone in supporting angiogenesis and neovascularization, we cannot exclude the possibility of chance leading to these changes/findings [29]. Nonetheless, these findings are intriguing because angiogenesis has been noted in MS plaques and in animal models of MS and angiogenesis can be closely intertwined with OPC maturation in neurovascular niches in the brain with some experts suggesting that angiogenesis may promote neuroregeneration [30]. Additionally, there is extensive evidence of the role of thyroid hormone in modulating immune cell function. Indeed, normal thyroid hormone signaling results in optimal macrophage functioning but either hypothyroidism or hyperthyroidism can lead to increased inflammatory responses and NLRP3 inflammasome activation [31]. However, caution is advised with interpreting the impact of liothyronine on the exploratory CSF biomarker findings given the small sample size and short duration of the study. A larger clinical trial would help determine whether these observed changes have a biological effect that is clinically meaningful.

There are several limitations worth mentioning. First, this trial included a small sample size and was short in duration, which only provides short-term safety and tolerability side effect profiles. Also, there was not a comparison group (placebo). This is expected for a phase 1 safety clinical trial with the recognition of possible different and/or more severe adverse events with longer-term exposure to a study drug. Second, the patient population enrolled was clinically heterogenous (relapsing and progressive patients with varying disease duration and DMTs), and the inclusion EDSS was restrictive. Future larger studies might identify whether age and/or disability status modifies the effects of liothyronine on remyelination and recovery. Finally, we did not include an imaging marker of remyelination, though such a marker remains incompletely validated and MRI is more commonly used in later stage clinical trials (e.g., phases 2 and 3).

This phase 1b study provides safety and tolerability data for using liothyronine in MS and could serve as first step towards another dose-titration study with examination of CNS penetration to find the highest tolerated dose for a Phase 2a proof of concept study. Moreover, a larger clinical trial could determine whether liothyronine can promote oligodendrogenesis and enhance remyelination in vivo, limit axonal degeneration, and improve function. Reassuringly, since clinical thyrotoxicosis did not occur in our study, it is reasonable to assume that larger trials could be successfully blinded. Future studies may also need to consider combination therapies that focus on different aspects of augmenting remyelination and repair in MS [32].
